# Antioxidant, antimicrobial, and theoretical studies of the thiosemicarbazone derivative Schiff base 2-(2-imino-1-methylimidazolidin-4-ylidene)hydrazinecarbothioamide (IMHC)

**DOI:** 10.1186/2191-2858-2-4

**Published:** 2012-02-02

**Authors:** Ahmed A Al-Amiery, Yasmien K Al-Majedy, Heba H Ibrahim, Ali A Al-Tamimi

**Affiliations:** 1 Biotechnology Division, Applied Science Department, University of Technology, Baghdad 10066, Iraq

**Keywords:** antibacterial, antioxidant, antifungal, creatinine, Schiff base, thiosemicarbazone

## Abstract

**Background:**

Adverse antimicrobial activities of thiosemicarbazone (TSC) and Schiff base derivatives have widely been studied by using different kinds of microbes, in addition different methods were used to assay the antioxidant activities using DPPH, peroxids, or ntrosyl methods. However, there are no studies describing the synthesis of TSC derived from creatinine.

**Results:**

In this study, 2-(2-imino-1-methylimidazolidin-4-ylidene)hydrazinecarbothioamide (IMHC) was synthesized by the reaction of creatinine with thiosemicarbazide. The novel molecule was characterized by FT-IR, UV-VIS, and NMR spectra in addition of the elemental analysis. The free radical scavenging ability of the IMHC was determined by it interaction with the stable-free radical 2,2"-diphenyl-1-picrylhydrazyl (or nitric oxide or hydrogen peroxide) and showed encouraging antioxidant activities. Density functional theory calculations of the IMHC performed using molecular structures with optimized geometries. Molecular orbital calculations provide a detailed description of the orbitals, including spatial characteristics, nodal patterns, and the contributions of individual atoms. Highest occupied molecular orbital-lowest unoccupied molecular orbital energies and structures are shown.

**Conclusions:**

IMHC shows considerable antibacterial and antifungal activities. The free radical scavenging activity of synthesized compound was screened for *in vitro *antioxidant activity.

## Background

Schiff-base compounds have been used as fine chemicals and medical substrates [1]. Azomethine group (-C = N-)-containing compounds, typically known as Schiff's bases, have been synthesized via condensation of primary amines with active carbonyls. It is well established that the biological activity of hydrazone compounds is associated with the presence of the active (-CO-NHN = C-) pharmacophore and these compounds form a significant category of compounds in medicinal and pharmaceutical chemistry with several biological applications that include antitumoral [2,3], antifungal [4-9], antibacterial [10,11], antimicrobial [12], and anthelmintic uses [13]. Schiff's base complexes play an important role in designing metal complexes related to synthetic and natural oxygen carriers [14,15]. Schiff bases (SBs) are important intermediates for the synthesis of some bioactive compounds such as ß-lactams [16-18], and employed as ligands for the complexation of metal ions [19]. SBs and their complexes are largely studied because they interested and important properties such as their ability to bind reversibly oxygen [20] redox systems in biological systems and oxidation of DNA [21].

Antioxidants are extensively studied for their capacity for protect organism and cell from damage that is induced by oxidative stress. Scientists in many different disciplines become more interested in new compounds, either synthesized or obtained from natural sources that could provide active components to prevent or reduce the impact of oxidative stress on cell [22,23].

The preparation of a 2-(2-imino-1-methylimidazolidin-4-ylidene)hydrazinecarbothioamide (IMHC) from thiosemicarbazide and creatinine is presented in this study. The structure established based on the extensive NMR spectroscopic studies. The microbial activities of IMHC and their *in vitro *antioxidant activities were also investigated. It was envisaged that these two active pharmacological molecules (thiosemicbazide and creatinine) if linked together would generate novel molecular templates, which are likely to exhibit interesting biological properties.

## Results and discussion

### Chemistry

#### UV/visible spectra

The UV-VIS of IMHC was recorded. The solution of IMHC in DMF exhibited two peaks at 255 and 322 nm (39215 and 31055 cm^-1^) which are attributed to π → π* or *n *→ π*.

#### FT-IR spectroscopy

The FT-IR spectra provide valuable information regarding the nature of functional group of IMHC. The appearance of a broad strong band in the IR spectra of IMHC in 3421 cm^-1 ^is assigned to N-H stretching vibrations of the primary amine group. The spectrum of IMHC shows two different -C = N bands at 1631 and 1618 cm^-1^.

**Scheme 1:**
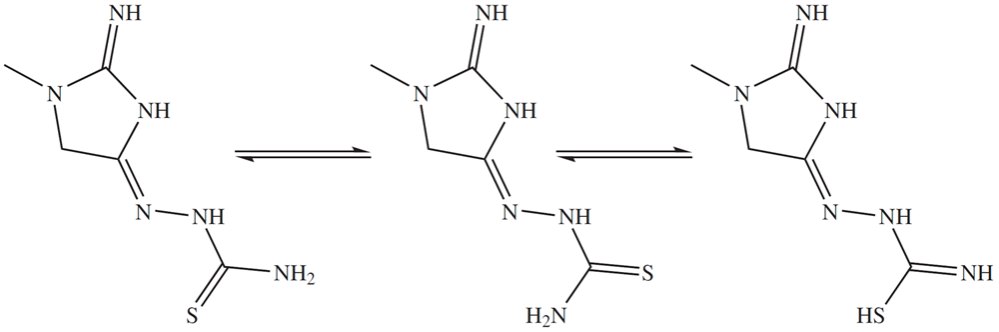
Tautomerization of thione.

Owing to the restricted rotation around the C = N bond, the IMHC may exist into two different geometric isomeric forms. The structure determination of one representative IMHC shows (Scheme 1) that the IMHC exists in thione form and corresponds to structure where the creatinine group is *cis *to the hydrazinic nitrogen across the C = N bond. The existence of the thione form predominantly in the solid state is demonstrated by the presence of two absorption bands at 1273.7 and 3421 cm^-1 ^belonging to the C = S and NH groups, respectively, and by absence of SH.

#### Density functional theory (DFT) studies

**Figure 1:**
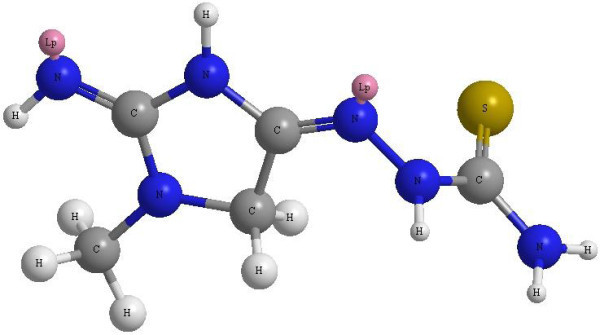
**Optimized 3D structure of the IMHC**.

DFT calculations of the IMHC (Figure 1) have been done using the optimized geometry molecular structures, Molecular orbital calculations provide a detailed description of orbitals including spatial characteristics, nodal patterns, and individual atom contributions. The energy of highest occupied molecular orbital (HOMO) of IMHC is -0.150240 Hartree, whereas the energy of lowest unoccupied molecular orbital (LUMO) of IMHC is 0.1102540 Hartree (Table 1). The lower value in the HOMO and LUMO energy gap explains the eventual charge transfer interaction taking place within the molecules.

**Table 1:** HOMO and LUMO energy

-0.150240 Hartree	-0.1102540 Hartree
	

### Pharmacology

#### Antibacterial activity

**Figure 2:**
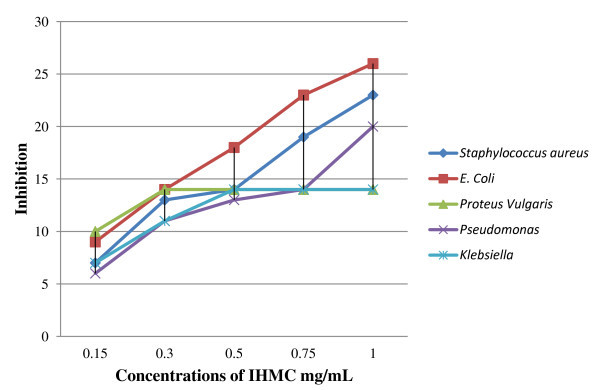
**The effect of test organism toward synthesized compound**.

The results of antibacterial activity study for IMHC indicated that the new molecule exhibited antibacterial activity against the studied bacteria at low and high concentrations. The increased activity of the synthesized compound can be explained electron delocalization over the whole molecule. This increases the lipophilic character of the molecule and favors its permeation through the lipoid layer of the bacterial membranes. The increased lipophilic character of this molecule seems to be responsible for it enhanced potent antibacterial activity. It may be suggested that this molecule deactivate various cellular enzymes, which play a vital role in various metabolic pathways of these microorganisms (Figure 2).

#### Antifungal activity

According to Overtone's concept of cell permeability, the lipid membrane that surrounds the cell favors the passage of only lipid-soluble materials, so lipophilicity is an important factor controlling the antifungal activity. Delocalization of π-electrons over the IMHC increased lipophilicity facilitates the penetration of the IMHC into lipid membranes, further restricting proliferation of the microorganisms. Although the exact biochemical mechanism is not completely understood, the mode of action of antimicrobials may involve various targets in the microorganisms.

• Interference with the synthesis of cellular walls, causing damage that can lead to altered cell permeability characteristics or disorganized lipoprotein arrangements, ultimately resulting in cell death.

• Deactivation of various cellular enzymes that play a vital role in the metabolic pathways of these microorganisms.

• Denaturation of one or more cellular proteins, causing the normal cellular processes to be impaired.

• Formation of a hydrogen bond through the azomethine group with the active centers of various cellular constituents, resulting in interference with normal cellular processes [24].

**Figure 3:**
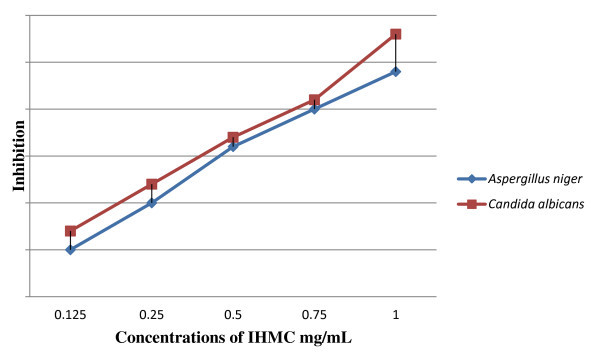
**The effect of tested fungi toward synthesized compound**.

*In vitro *antifungal screening effects of the investigated compound was tested against some fungal spices (*Aspergillus niger and Candida albicans*). It was found to that the new compound exhibits antifungal activity against *C. albicans *more than *A. niger *(Figure 3).

### Antioxidant activity

**Figure 4:**
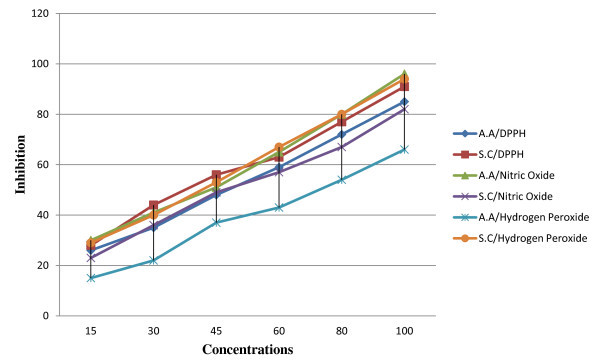
**The effect of synthesized compound towrd DPPH, nitric oxide and hydrogen peroxide**.

The role of antioxidant is to remove free radical. One important mechanism through which this is achieved is by donating hydrogen to free radicals in its reduction to an unreactive species. Addition of hydrogen would remove the odd electron feature which is responsible for radical reactivity. The hydrogen-donating activity, measured using DPPH (1,1-diphenyl-2-picrilhydrazyl) radicals as hydrogen acceptor, showed that a significant association could be found between the concentration of novel molecule and percentage of inhibition (Figure 4).

## Experiment

### Chemistry

#### Matierials

All chemical used were of reagent grade (supplied by either Merck or Fluka) and used as-received. The FTIR spectra were recorded as KBr disc on FTIR 8300 Shimadzu Spectrophotometer. The UV-Visible spectra were measured using Shimadzu UV-Vis. 160 A spectrophotometer. Proton NMR spectra were recorded on Bruker - DPX 300 MHz spectrometer with TMS as internal standard. Elemental microanalysis was carried out using C.H.N elemental analyzer model 5500-Carlo Erba instrument.

### Synthesis of IMHC

**Scheme 2:**
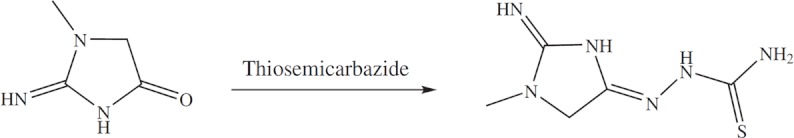
The synthesis of IMHC.

This mixture of hot ethanolic solution of thiosemicarbazide (1.82 g, 0.02 mol) and creatinine (2-imino-1-methylimidazolidin-4-one) (2.26 g, 0.02 mol) was refluxed with stirring for 3 h. The completion of the reaction was confirmed by the TLC. The reaction mass was degassed on a rotatory evaporator, over a water bath. Thiosemicarbazone filtered, washed with cold EtOH, and dried under vacuum over P_4_O_10_. Yield, 70; M.P. 153°C; light brown. Proton NMR **(**1.8(1H) for NH, s. 2.2(3H) for CH_3_, s. 2.7(2H) for CH_2_, 8 for NH, 9.1 for NH, 10.9 for NH_2_). Element chemical analysis data were C, 32.25(31.91); H, 5.41(5.11); N, 45.13(44.74), and the reaction equation was shown in Scheme 2.

## Pharmacology

### Antimicrobial activities

#### Antibacterial activity

The biological activity of the new IMHC was studied against selected types of bacteria which included positive bacteria (*Staphylococcus aureus*), and gram negative bacteria (*Escherichia coli*, *Klebsiella pneumoniae*, *Proteus vulgaris, Pseudomonas aeruginosa*), in brain hart broth agar media, which is used DMF as a solvent and as a control for the disc sensitivity test [25]. This method involves the exposure of the zone of inhibition toward the diffusion of microorganism on agar plate. The plates were incubated for 24 h, at 37°C. The antimicrobial activity was recorded as any area of microbial growth inhibition that occurred in the diffusion area.

#### Antifungal activities

IMHC was screened for it antifungal activity against *A. niger *and *C. albicans *in DMSO by serial plate dilution method using sabourand agar media. Normal saline was used to make a suspension of corresponding species. Twenty milliliters of agar media was poured in each Petri dish. Excess suspension was decanted and the plates were dried by placing in an incubator at 37°C for 1 h [15]. The fungal zone of inhibition values is given in Figure 3. The nutrient broth was inoculated with approximately 1 × 10^5 ^cfu/mL. The cultures were incubated for 48 h at 35°C and the growth was monitored.

*Hint*: Sabourand agar media were prepared by dissolving peptone (1 g), D-glucose (4 g), and agar (2 g) in distilled water (100 mL) and adjusting pH to 5.7.

#### Antioxidant studies

##### (2,2-diphenyl-1-picrylhydrazyl) radical scavenging activity

The DPPH radical scavenging activities of the test IMHC were evaluated [26]. Initially, 0.1 mL of IMHC at concentration of 250, 500, 750, and 1000 μg/mL was mixed with 1 mL of 0.2 mM DPPH that was dissolved in methanol. The reaction mixture was incubated in the dark for 20 min at 28°C. The control contained all reagents without the sample while methanol was used as blank. The DPPH radical scavenging activity was determined by measuring the absorbance at 517 nm using the UV-Vis spectrophotometer. The DPPH radical scavenging activity of ascorbic acid was also assayed for comparison. The percentage of DPPH radical scavenger was calculated using Equation 1.

$$\text{Scavenging effects}\left(\%\right)=\frac{A_{0}-A_{1}}{A_{0}}\times 100$$

where *A*_0 _is the absorbance of the control reaction and *A*_1 _is the absorbance in the presence of the samples or standards.

##### Nitric oxide scavenging activity

Sodium nitroprusside in aqueous solution at physiological pH generates nitric oxide spontaneously; it interacts with oxygen to produce nitrite ions, which can be estimated by the use of GriessIllosvoy reaction [27,28]. In this investigation, GriessIllosvoy reagent was modified using naphthylethylenediaminedihydrochloride (0.1% w/v) instead of 1-naphthylamine (5%). The reaction mixture (3 mL) containing sodium nitroprusside (10 mM, 2 mL), phosphate buffer saline (0.5 mL), and IMHC (250, 500, 750, and 1000 μg/mL) or standard solution (0.5 mL) was incubated at 25°C for 150 min. After the incubation, 0.5 mL of the reaction mixture containing nitrite was pipetted and mixed with 1 mL of sulfanilic acid reagent (0.33% in 20% glacial acetic acid) and allowed to stand for 5 min for completing diazotization. Then, 1 mL of naphthylethylenediaminedihydrochloride (1%) was added, mixed, and allowed to stand for 30 min. A pink-colored chromophore was formed in diffused light. The absorbance of these solutions was measured at 540 nm against the corresponding blank. Ascorbic acid was used as standard. Nitric oxide percentage scavenging activity was then calculated using Equation 1.

##### Hydrogen peroxide scavenging activity

A solution of hydrogen peroxide (40 mM) was prepared in phosphate buffer (pH 7.4). Different concentrations (250, 500, 750, and 1000 μg/mL) of IMHC (or ascorbic acid) were added to a hydrogen peroxide solution (0.6 mL, 40 mM). Absorbance of hydrogen peroxide at 230 nm was determined after 10 min against a blank solution containing phosphate buffer without hydrogen peroxide [29]. Hydrogen peroxide percentage scavenging activity was then calculated using Equation 1.

#### DFT

All quantum chemical calculations were performed using the DFT in the methodology. DMol3 model was employed to obtain quantum chemical parameters and optimization of the molecule geometry.

## Competing interests

The HOMO-LUMO explain the presence of an isolated state within the electronic structure of the compound, which is responsible for the intense blue-violet emission. It is well-known that orbital energy differences strongly overestimate actual excitation energies, and either configuration interaction or time-dependent treatments are needed to model the energetics of the electronic excitations. Nevertheless, the orbital energies provide a useful qualitative description [29].
